# Triplets, with Dropsy of the Amnion

**Published:** 1871

**Authors:** Edwin S. Ray

**Affiliations:** Atlanta, Ga.


### *Triplets, with Dropsy of the Amnion.* By EDWIN S. RAY, M. D.

[Read before the Atlanta Academy of Medicine.]

In November, 1870, Dr. J. G. Westmoreland and myself were requested by Dr. Gilbert, of this city, to go with him to a neighboring town, where he desired professional consultation in the case of a married lady, aged 26 years. She was the mother of two children, the younger being eighteen months old.

Six months previous to our visit, her health, which had always been good, commenced to fail and continued to grow worse until the time above mentioned. She believed herself dropsical. Upon examination we found her abdomen greatly distended; so much so, indeed, as to prevent locomotion. Her breathing was labored and painful, in consequence of pressure against the diaphragm, by the great effusion which we believed to be in the peritoneal cavity. Fluctuation was very distinct at every point, except immediately over the symphibis pubis. She did not think herself pregnant; nor did we, during the examination then made, discover any reason for believing her in that condition. Fearing, however, that she might be, and judging it advisable to await further development in the case, she, at our suggestion, was placed upon the train, which ran within a few yards of her residence, and brought to the city of Atlanta, where we could better watch her condition.

Shortly after her arrival, Dr. W. F. Westmoreland joined us in further consultation. In the meantime, the enormous enlargement of her abdomen seemed to increase. Her suffering became more agonizing. Full doses of morphine were administered. While we listened in vain for the fœtal heart, we felt, for the first time, a slight movement in the lower portion of her abdomen, which made us believe that she was probably pregnant. Examinations per vaginam threw no additional light on the case, except that the neck of the womb was found somewhat enlarged and shortened, and the os inclined to yield to the pressure, whatever it might be. This condition, coupled with the facts, that little or no fluctuation could be detected over the symphibis pubis, and that a movement in the proper locality had become certain, made us believe that our patient not only had an enormous quantity of fluid in the peritoneal cavity, but that she was also pretty far advanced in pregnancy. Neither the size nor shape of the womb could be determined, though we, for that purpose, did everything possible.

It was late in the afternoon that we thus agreed. Something had to be done, and that quickly. The poor woman was begging most piteously for relief. Ought we to put a trochar into the peritoneal cavity and draw off the effusion that was pressing down the uterus? We thought so; and after giving the patient a strong anodyne, assured her that early next morning she should have relief. Before midnight came I was called by her husband, who informed me that his wife had just given birth to a child. I was urged to go at once to her assistance. In a few minutes I was in her room, and found that her mother had taken away a female child of six months. I at once attempted to bring away the placenta, and in doing so found the distension of the abdomen apparently as great as ever. Passing my finger along the cord, a membranous obstruction completely spanning and as completely dividing the lower part of the womb, was felt. At this juncture I desired and obtained the co-operation of Dr. W. F. Westmoreland. While making his examination, and trying to ascertain positively the nature of the difficulty, the membrane gave way, followed by a rapid discharge of between *four and five gallons of water*. Again introducing his finger, the feet of another child were found protruding through the rent in the membrane. Within ten minutes he (for it was a male child) was born, and was so dropsical as to be three times as large as his sister, who had just preceded him. Of course we now knew that the extraordinary enlargement of her abdomen had been produced by DROPSY

of the Amnion, and not by an effusion into the peritoneal cavity, as we had supposed. True, there might have been both. In this instance, however, such was not the case. Just here we were disposed to congratulate the lady, so much relieved, upon her good luck, and to assure her that all would be right very soon, when membrane No. 3 gave way, and another gush of water (between one and two pints in quantity) and another male child, smaller than his sister, were before us. The female child lived about three hours. The males were born dead. The mother recovered perfectly.

My main object in reporting the above case, which was so interesting to the attending physicians, is to call the attention of the younger and less experienced members of our profession to the impossibility of clearly distinguishing, in many cases, Amniotic from Peritoneal Dropsy. Here, for instance, the womb was so enormously distended (*not only by three children but by Five Gallons of Water*) as to give the abdomen the characteristic appearance of Ascites. Excepting myself, the physicians engaged in the consultation, were gentlemen of much experience. We did everything possible to ascertain, beyond doubt, her trouble, her danger, and our duty. We unanimously agreed in the belief that she was pregnant, but we were more certain that she had peritoneal dropsy, and that nothing short of tapping would probably give relief. In this connection, it may not be amiss to state that a few cases are published, in which Accoucheurs of considerable distinction (mistaken in their conclusions, as we were), have actually plunged the trochar through the abdominal wall into the uterus, and into the fœtus.

I would add, in conclusion, that it was not my intention, in the beginning, to express an opinion as to the most frequent cause of Amniotic Dropsy. I had purposed making an examination of the Amnion, on the morning after delivery, but found it had been destroyed.

